# Application of a Cloud Model-Set Pair Analysis in Hazard Assessment for Biomass Gasification Stations

**DOI:** 10.1371/journal.pone.0170012

**Published:** 2017-01-11

**Authors:** Fang Yan, Kaili Xu

**Affiliations:** School of Resources and Civil engineering, Northeastern University, Shenyang, Liaoning, P. R. China; Southwest University, CHINA

## Abstract

Because a biomass gasification station includes various hazard factors, hazard assessment is needed and significant. In this article, the cloud model (CM) is employed to improve set pair analysis (SPA), and a novel hazard assessment method for a biomass gasification station is proposed based on the cloud model-set pair analysis (CM-SPA). In this method, cloud weight is proposed to be the weight of index. In contrast to the index weight of other methods, cloud weight is shown by cloud descriptors; hence, the randomness and fuzziness of cloud weight will make it effective to reflect the linguistic variables of experts. Then, the cloud connection degree (CCD) is proposed to replace the connection degree (CD); the calculation algorithm of CCD is also worked out. By utilizing the CCD, the hazard assessment results are shown by some normal clouds, and the normal clouds are reflected by cloud descriptors; meanwhile, the hazard grade is confirmed by analyzing the cloud descriptors. After that, two biomass gasification stations undergo hazard assessment via CM-SPA and AHP based SPA, respectively. The comparison of assessment results illustrates that the CM-SPA is suitable and effective for the hazard assessment of a biomass gasification station and that CM-SPA will make the assessment results more reasonable and scientific.

## Introduction

The energy crisis has confused mankind for several decades, especially in many low- and middle-income countries [1]. To solve this problem, the development of renewable energy is significant. Biomass has tremendous potential in solving future shortages of energy as renewable resources [2], and it has undergone rapid development in recent years [3–5]. Likewise, as a middle-income developing country, biomass energy has also recently undergone rapid development in China [6,7]. Utilization of biomass resources is multifarious, including biodiesel [8], biomass to liquid (BTL, [9]), biomass gasification [10], and so on. However, biomass gasification is considered to be a crucial utilization alternative [11]. In recent years, biomass gasification stations have been constructed and massively put into service in rural areas of China. The agriculture wastes are converted into green energy *via* biomass gasification. Herein the biomass gas is produced by the burning of agriculture wastes under the condition of insufficient oxygen [12], thus the produced biomass gas contains hydrogen (H_2_), carbon monoxide (CO), and methane (CH_4_) which are flammable and explosive while CO is poisonous [13,14]. Because the produced biomass gas and biomass materials are flammable and the biomass gas has explosive and poisonous characteristics, hazard factors exist in biomass gasification stations. As a result, to conduct risk control and safety management for a biomass gasification station, hazard assessment is necessary.

Hazard assessment can also be called risk assessment, and it's widely used in the prediction and prevention of accidents. Hazard assessment includes various methods, such as a fire and explosion index (F&EI, [15,16]), a fuzzy risk assessment [17,18], and an analytic hierarchy process (AHP, [19,20]), among others. However, the set pair analysis (SPA) proposed by Zhao [21] can also be used in hazard assessment. SPA is a multifunctional assessment method that is simple in concept and easy to apply [22], and it is a systematic analysis method that deals with uncertain problems; it is also an uncertainty theory that integrates certainty and uncertainty [23]. SPA is practical in various fields and can be used in the investigation of leaking water from a dam [24], quality assessment of groundwater [25], evaluation of surrounding rock stability [26], hazard assessment of debris flow and landslides [22,27], assessment of a water resources system [28], comprehensive risk assessment of floods [23], safety assessment of thermal power plants [29], risk assessment of water pollution sources [30], prediction analysis of integrated carrying capacity [31], and so on. In SPA, one of the cores is the confirmation of assessment index weight. Several methods are involved in the confirmation of index weight. The information entropy method (IEM) can be utilized to confirm the index weight [22,24,25,30]. AHP can also be used to confirm the index weight [32,33]. Furthermore, fuzzy methods can be coupled with AHP, and the fuzzy AHP will make the index weight more reasonable [23]. The index weights confirmed by the above methods have a common characteristic, i.e., the value of the index weight is a single numerical value. In other words, the SPA results will be greatly influenced by the value of the index weight. While the index weight is confirmed via expert judgment, the subjective consciousness of experts will lead to results. Therefore, a cloud model (CM) can be employed to confirm the index weight due to its characteristics of randomness and fuzziness [34]. CM was proposed by Li [35] to handle uncertainty problems. CM is used to make a conversion between qualitative concepts and quantitative values; then, the randomness and fuzziness of uncertainty can be fully reflected by CM [36]. It has tremendous application in various fields. Liu [34] conducted a comprehensive stability evaluation of rock slopes using a cloud model-based approach; in their study, various factors of slope stability were analyzed based on their cloud membership. Zhang used a cloud model-based method to analyze the accelerated life test data [37]; due to the random uncertainties existing in the testing data, a multi-rule-based cloud reasoner was proposed and the relationship between the uncertain stress level and the means of sample lifetimes was derived. Parhoudeh [38] took advantage of the cloud model to establish a novel stochastic framework for the handling of the uncertainty effects in the optimal operation of micro-grids. CM can also be used in topology optimization [39], risk assessment [40], image segmentation [41] and so on. Moreover, CM is useful in dealing with uncertain linguistic variables [42,43]. In Wu's study [44], SPA was coupled with CM to create a set pair fuzzy decision method. Based on their method, the qualitative linguistic description of identity, discrepancy and contradistinction by experts can be converted into a quantitative value so that the quantitative SPA can be made. However, in the process of SPA, the index weight is confirmed by expert judgment; therefore, the linguistic variables of expert judgment can also be handled by CM so that the index weight can be more reasonable and scientific.

This study proposes a novel hazard assessment method based on the cloud model-set pair analysis (CM-SPA). In regard to the proposed method, the index weight is confirmed by the CM and the cloud weight is extracted. In contrast with other index weight confirmation methods, the cloud weight is denoted by the cloud descriptors rather than a constant value. Then, the connection degree (CD) of SPA is replaced with the cloud connection degree (CCD) based on the cloud weight. Meanwhile, the calculation algorithm of CCD is worked out in this paper. Finally, two biomass gasification stations in Shenyang City, Liaoning Province, northeast China, are made hazard assessment by CM-SPA and AHP based SPA, respectively.

## Methodology

### Set Pair Analysis

As an uncertainty theory proposed by Zhao [21], SPA is a systematic methodology that deals with the integration of certainty and uncertainty problems. The relationship of certainty and uncertainty discussed in SPA is analyzed based on identity, discrepancy and contradistinction [25,28]. Let two sets *A* and *B* be a pair set *H* = (*A*, *B*). Meanwhile, assume the total number of characteristics in *H* to be *N*. Then, assume the amounts of identical characteristics to be *S*, the amounts of discrepant characteristics to be *P*, and the amounts of contrary characteristics to be *F*. Based on the definition of SPA [21,31], the relation of *N*, *S*, *P* and *F* is *N* = *S*+*P*+*F*. Then, CD is used to describe the connection of *H* = (*A*, *B*); the calculation algorithm of the connection degree is shown below (Eq 1, [21]):

$$\varphi (H)=\frac{S}{N}+\frac{F}{N}i+\frac{P}{N}j=a+bi+cj$$ (1)

where *φ*(*H*) denotes the CD of *H* and *a*, *b* and *c* denote the identity degree, discrepancy degree and contradistinction degree, respectively. *i* and *j* are the discrepancy coefficient and the contradictory coefficient, respectively. It is defined that *i* is within [-1, 1], the value of *j* is 1, and the relation of *a*, *b* and *c* is *a*+*b*+*c* = 1.

Eq 1 shows the calculation of the CD of the basic SPA model. Moreover, the discrepancy degree can be divided into multiple grades and the model can be extended to the general form, called the *m*-element model. The CD of an *m*-element SPA is calculated by solving Eq 2 [26].

$$\varphi =a+\sum_{n=1}^{m-2}b_{n}i_{n}+cj$$ (2)

where *b*_*n*_ denotes different grades of the discrepancy degree; *i*_*n*_ denotes different grades of the discrepancy coefficient.

For the CD of index *k*, the values of the identity degree, discrepancy degree and contradistinction degree are set as '0' or '1'. If the assessment value belongs to the hazard grade, the value is set as '1'; otherwise, the value is set as '0'. In general, the index weight is in the focus of SPA. For the traditional SPA, the index weight is a constant; the final hazard assessment results, i.e., the total CD, is calculated by solving Eq 3 [23–25].

$$\varphi =\sum_{k=1}^{r}\varphi_{k}\omega_{k}$$ (3)

where *φ* denotes the total CD, *ω*_*k*_ denotes the weight of index *k*, *r* denotes the amounts of indices, and *φ*_*k*_ denotes the CD of index *k*.

In the hazard assessment based on SPA, the assessment results are confirmed by the maximal connection degree principle. For a sample *l* with the *m*-element model, the corresponding amounts of the hazard grades are also set to *k*. Assume that the total CD for sample *l* is *φ*_*l*_ = *a*+*b*_1_*i*_1_+*b*_2_*i*_2_+…+*b*_*k*-2_*i*_*k*-2_+*cj*; if it is satisfied with the condition *d* = max{*a*,*b*_1_,*b*_2_,…,*b*_*k*-2_,*c*}, then the hazard grade of sample *l* can be confirmed as the corresponding grade of *d*.

### Cloud Model

In general, each index of the SPA was not the same in the hazard assessment; they had different weights. The weight of an index can be confirmed by AHP, IEM, and so on. In this study, CM was employed to confirm the weight of indices in SPA; then, the CM-SPA was used to conduct a hazard assessment of a biomass gasification station.

For the given universe of discourse *U*, *C* is the corresponding concept of *U* and *x* ∈ *U* is the definite parameter, which is also a random occurrence of *C*. If the membership *μ*(*x*) ∈ [0, 1] of *x* to *C* is a random number with steady tendency, then the distribution of *x* in *U* is called the cloud and each *x* is called the cloud drop (Eq 4).

μ:U→[0,1] ∀x∈U x→μ(x) (4)

In the CM, the digital characteristics are defined as *C* = (*Ex*, *En*, *He*) to describe the uncertainty. Among these digital characteristics, *Ex*, *En* and *He* denote the expected value, entropy and hyper entropy, respectively. *Ex* is the expected value of the cloud drop in the distribution of universe of discourse *U*. Generally, it is the point that can represent the qualitative concept. *En* denotes the uncertainty measurement of the qualitative concept; it is confirmed by the randomness and fuzziness of concepts together. The dispersion degree of cloud drops is reflected by *En*, which also determines the certainty of cloud drops. *He* is the entropy of *En*; it reveals the uncertainty measurement of *En*. Likewise, *He* can be called the second-order entropy. For the commonsense concept, *He* is smaller when the acceptance degree is higher. *He* will be bigger if the concept cannot reach an agreement. Let *U* be the quantitative universe of discourse and *C* = (*Ex*, *En*, *He*) be the qualitative concept in *U*. If the quantitative value of *x* (*x* ∈ *U*) is a random instantiation of *C*, *x* satisfies *x* ~ *N*(*Ex*, *En*'^2^). Meanwhile, *En*' is also a random instantiation and satisfies *En*' ~ *N*(*En*, *He*^2^). Then, the certainty of *x* to *C* satisfies Eq 5.

$$\mu =e^{-\frac{{\left(x-Ex\right)}^{2}}{2{\left(En\prime \right)}^{2}}}$$ (5)

**Fig 1:**
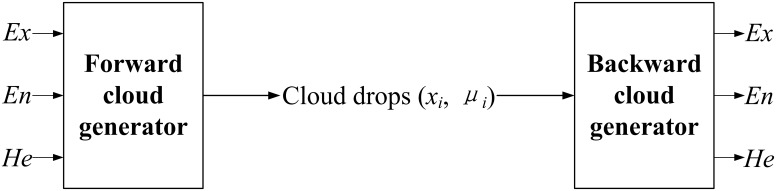
Cloud generator.

The distribution of cloud drop *x* in universe of discourse *U* is called the normal cloud. Then, cloud drops are generated by a forward cloud generator. As shown in Fig 1, when the cloud descriptors *C* = (*Ex*, *En*, *He*) are given, the cloud drops *P* = (*x*_*i*_, *μ*_*i*_) will be obtained to represent the qualitative concepts. On the contrary, if the cloud drops *P* = (*x*_*i*_, *μ*_*i*_) are known, then the cloud descriptors *C* = (*Ex*, *En*, *He*) can be obtained by the backward cloud generator [34,45].

**Fig 2:**
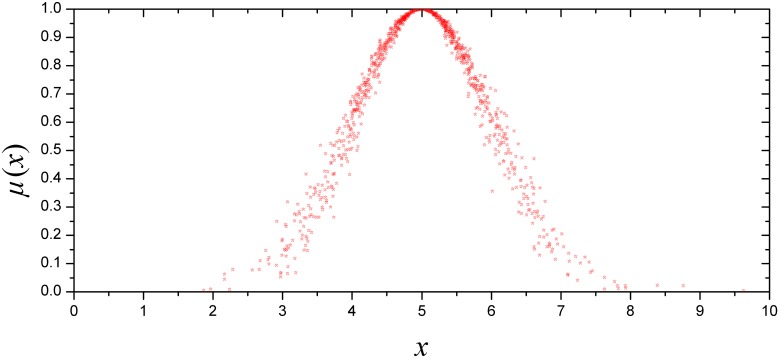
An example of a normal cloud.

Assume a normal cloud and let *Ex*, *En* and *He* be 5, 1 and 0.1, respectively. Meanwhile, the amounts of cloud drops are set as 1000. Then, the normal cloud is generated by the forward cloud generator (Fig 2). Regarding the cloud drops that contribute to the qualitative concepts, they are mainly focused on [*Ex*-3*En*, *Ex*+3*En*]. According to the different contributions of these cloud drops, they are divided into basic elements, peripheral elements and more peripheral elements, which belong to [*Ex*-*En*, *Ex*+*En*], [*Ex*-2*En*, *Ex*-*En*]∪[*Ex*+*En*, *Ex*+2*En*] and [*Ex*-3*En*, *Ex*-2*En*]∪[*Ex*+2*En*, *Ex*+3*En*], respectively. The contribution of cloud drops outside [*Ex*-3*En*, *Ex*+3*En*] can be neglected. The definition is called the '3*En* rule' [36,46].

For the representation of concepts in CM, the confusion degree is introduced (Eq 6). It is used to measure the consensus degree of concepts; if the value of the confusion degree is equal to or greater than 1, then the cloud will be 'fog'. In other words, the concepts cannot reach consensus and need to be given again [35,36].

$$\text{confusion degree}=\frac{3He}{En}$$ (6)

### Cloud Model-Set Pair Analysis

In this paper, the CM was employed to confirm the index weight in SPA, and the new type of weight is called the cloud weight. Because the index weight in SPA is confirmed using linguistic variables [42], the linguistic variables are converted into a definite value by traditional methods. However, the definite value cannot reflect the uncertainty of linguistic variables. As a result, the hazard assessment results will be greatly influenced by the value of the index weight; this makes the final hazard assessment results inaccurate. Hence, cloud weight takes the place of the traditional weight for indices; meanwhile, the hazard assessment results are also expressed by the cloud descriptors. Then, the randomness and fuzziness of linguistic variables can be fully reflected and the hazard assessment results will be more reasonable. The steps of CM-SPA are shown below.

1. Experts judge each index by using the linguistic variables. The judgment criterion is shown in Table 1.
  Then, the judgment results are normalized (Eq 7).
  $$X_{kl}=\frac{x_{kl}}{\sum_{k=1}^{r}x_{kl}}$$ (7)
  where *x*_*kl*_ denotes the judgment of index *k* by expert *l*, *r* denotes the amounts of indices, and *X*_*kl*_ denotes the normalized value of *x*_*kl*_.
2. After the judgments for the index weight are obtained, these qualitative linguistic variables are converted into quantitative values, i.e., the cloud descriptors *Ex*, *En* and *He* (Eq 8) [36,37,46].
  $$\left\{ \begin{array}{l} Ex_{k}=\frac{1}{N}\sum_{l=1}^{N}X_{kl}\\ En_{k}=\sqrt{\frac{\pi }{2}}\times \frac{1}{N}\sum_{l=1}^{N}\left|X_{kl}-Ex_{k}\right|\\ He_{k}=\sqrt{\left|\frac{1}{N-1}\sum_{l=1}^{N}{\left(X_{kl}-Ex_{k}\right)}^{2}-{\left(En_{k}\right)}^{2}\right|} \end{array}\right.$$ (8)
  Then, the cloud weight of index *k* is displayed as the cloud descriptors *ω*(*C*)_*k*_ = (*Ex*_*k*_, *En*_*k*_, *He*_*k*_).
3. The hazard assessment results by SPA focus on the cloud weight of the index afterwards. As previously mentioned, Eqs 2 and 3 can be re-written as Eq 9 to calculate the total CD based on CM, called the CCD.
  $$\begin{array}{ll} \varphi (C) & =\varphi_{1}⊗\omega {\left(C\right)}_{1}⊕\varphi_{2}⊗\omega {\left(C\right)}_{2}⊕\ldots ⊕\varphi_{k}⊗\omega {\left(C\right)}_{k}\\ & =a_{1}\omega {\left(C\right)}_{1}⊕a_{2}\omega {\left(C\right)}_{2}⊕\ldots ⊕a_{k}\omega {\left(C\right)}_{k}\\ & +\left(b_{1,1}\omega {\left(C\right)}_{1}⊕b_{2,1}\omega {\left(C\right)}_{2}⊕\ldots ⊕b_{k,1}\omega {\left(C\right)}_{k}\right)i_{1}\\ & +\left(b_{1,2}\omega {\left(C\right)}_{1}⊕b_{2,2}\omega {\left(C\right)}_{2}⊕\ldots ⊕b_{k,2}\omega {\left(C\right)}_{k}\right)i_{2}+\ldots \\ & +\left(b_{1,m-2}\omega {\left(C\right)}_{1}⊕b_{2,m-2}\omega {\left(C\right)}_{2}⊕\ldots ⊕b_{k,m-2}\omega {\left(C\right)}_{k}\right)i_{m-2}\\ & +\left(c_{1}\omega {\left(C\right)}_{1}⊕c_{2}\omega {\left(C\right)}_{2}⊕\ldots ⊕c_{k}\omega {\left(C\right)}_{k}\right)j \end{array}$$ (9)
  For Eq 9, the calculation involves the summation of *Ex*, *En* and *He*. The calculation algorithm is shown below (Eq 10) [43].
  $$\left\{ \begin{array}{l} Ex_{s}=Ex_{1}⊕Ex_{2}⊕\ldots ⊕Ex_{k}=Ex_{1}+Ex_{2}+\ldots +Ex_{k}\\ En_{s}=En_{1}⊕En_{2}⊕\ldots ⊕En_{k}=\sqrt{En_{1}^{2}+En_{2}^{2}+\ldots +En_{k}^{2}}\\ He_{s}=He_{1}⊕He_{2}⊕\ldots ⊕He_{k}=\sqrt{He_{1}^{2}+He_{2}^{2}+\ldots +He_{k}^{2}} \end{array}\right.$$ (10)
  where *Ex*_*s*_, *En*_*s*_ and *He*_*s*_ denote the summation of the expected value, entropy and hyper entropy, respectively.
4. As shown in Eq 9, the final hazard assessment results, i.e., the CCD *φ*(*C*), consist of several normal clouds. Generally, if the SPA is an *m*-element model, there are *m* normal clouds displayed in the final hazard assessment results. However, in some cases, the value of the identity degree, discrepancy degree or contradistinction degree may be '0'; thus, the corresponding normal cloud is non-existent. After that, the hazard grade is confirmed by the descriptors of these normal clouds based on the maximal connection degree principle and the '3*En* rule' as previously mentioned.

**Table 1:** Judgment criterion of experts for index weight.

Linguistic Variables Level	Value Range
Very important	(8, 10]
Important	(6, 8]
Middle important	(4, 6]
Unimportant	(2, 4]
Very unimportant	(0, 2]

### Case Study

In this study, no specific permissions were required for the locations introduced. Because these locations are public area and our activities were permitted by Shenyang Municipality. We can ensure that the field studies did not involve endangered or protected species. Afterwards, two biomass gasification stations were introduced to conduct a hazard assessment using CM-SPA. The two stations' names were Huangtukan Village biomass gasification station and Yanjia Village biomass gasification station (hereafter referred to as 'Huangtukan station' and 'Yanjia station', respectively). Huangtukan station and Yanjia station are located in Huangtukan Village, Liaozhong District, Shenyang City, Liaoning Province, northeast China and Yanjia Village, Hunnan District, Shenyang City, Liaoning Province, northeast China, respectively. Huangtukan station is located at 122.767°E, 41.718°N; Yanjia station is located at 123.750°E, 41.996°N.

### Confirmation of Indices and Calculation of Cloud Weight

In a biomass gasification station, the hazard mainly comes from the biomass materials used to make biomass gas and the produced biomass gas. The produced biomass gas contains hydrogen (H_2_), carbon monoxide (CO), and methane (CH_4_) [47]. Thus, the biomass materials and biomass gas are flammable and the biomass gas has explosive characteristics. Meanwhile, the CO in biomass gas makes it poisonous. As a result, the hazard of biomass gasification contains fire, explosion and poisoning. In this study, 6 corresponding hazard assessment indices were introduced based on the immediate causes of fire, explosion and poisoning [48–50]: biomass gas production rate (*k*_1_), volume fraction of CO (*k*_2_), lower explosive limit of biomass gas (*k*_3_), artificial ventilation atmosphere (*k*_4_), pressure relief ratio (*k*_5_) and quantity of biomass materials (*k*_6_). However, many other assessment indices are involved into the hazard assessment for biomass gasification stations, such as the tar, human error, weather factors and so on. There may be large numbers of the assessment indices, whereas they aren't immediate causes. Thus the hazard assessment for biomass gasification stations made in this study can be regarded as a specific hazard assessment with respect to the most critical hazards. In regard to these introduced indices, 10 experts were invited to make judgments regarding their importance based on Table 1 while introductions of these experts were shown in Table 2; the cloud descriptors *Ex*, *En* and *He* were calculated by using Eq 7 through Eq 8. However, some judgments will not meet the objective fact due to the mistakes may made by some experts. For example, a general judgment of the importance for index *k*_1_ should be ‘middle important’ or ‘important’. If the judgment made by some expert was ‘very unimportant’, it meant that the judgment deviated from the objective fact, and experts cannot reach consensus. As previously mentioned, the generated cloud will be ‘fog’ when judgments of experts cannot reach consensus, that is to say, some judgments deviated from the objective fact. Then the ‘fog’ cannot be used to make assessment using CM [35,36]. Hence, the confusion degree was employed to check the validity of the judgment results [35,36], that is to say, whether the judgments will meet the objective fact of the biomass gasification or not should be checked. As a result, the cloud descriptors *En* and *He* should satisfy the condition that the value of the confusion degree must be less than 1. If the condition was not satisfied, then the judgments of experts needed to be made again until the condition was satisfied. The judgment results and cloud weight of each index are shown in Tables 3 and 4, respectively.

**Table 2:** Introduction of the experts.

	Professional position	Education background	Experience (years)
Expert 1	Student	Master	4
Expert 2	Student	PhD	7
Expert 3	Worker	Junior college	18
Expert 4	Worker	High School	30
Expert 5	Engineer	Bachelor	17
Expert 6	Engineer	Master	15
Expert 7	Engineer	Bachelor	23
Expert 8	Professor	PhD	29
Expert 9	Professor	PhD	32
Expert 10	Professor	PhD	27

**Table 3:** Results of experts’ judgments regarding the importance of hazard assessment indices.

	*k*_1_	*k*_2_	*k*_3_	*k*_4_	*k*_5_	*k*_6_
Expert 1	6	1	10	8	4	2
Expert 2	5	2	7	7	3	2
Expert 3	6	2	9	7	5	2
Expert 4	7	1	8	8	3	3
Expert 5	8	2	10	7	3	1
Expert 6	5	3	8	9	4	1
Expert 7	6	2	8	10	5	2
Expert 8	5	2	7	9	3	1
Expert 9	8	3	10	9	4	2
Expert 10	7	1	8	9	3	3

**Table 4:** Cloud weight of each index.

Index	Cloud Weight (*ω*(*C*))
Biomass gas production rate (*k*_1_)	(0.2053, 0.0297, 0.0092)
Volume fraction of CO (*k*_2_)	(0.0622, 0.0226, 0.0054)
Lower explosive limit of biomass gas (*k*_3_)	(0.2776, 0.0258, 0.0072)
Artificial ventilation atmosphere (*k*_4_)	(0.2722, 0.0345, 0.0032)
Pressure relief ratio (*k*_5_)	(0.1206, 0.0232, 0.0052)
Quantity of biomass materials (*k*_6_)	(0.0622, 0.0231, 0.0073)

### Classification of Hazard Grade for Assessment Index

In this study, the SPA was set as a 5-element model; therefore, the general type of CD was *φ* = *a*+*b*_1_*i*_1_+*b*_2_*i*_2_+*b*_3_*i*_3_+*cj* and the 5 corresponding hazard grades were named very low hazard (I), low hazard (II), middle hazard (III), high hazard (IV) and very high hazard (V). Index *k*_1_ denotes the ability of biomass gas production; its unit is m^3^/h. Index *k*_2_ denotes the volume fraction of CO in the produced biomass gas; its unit is %. Index *k*_3_ denotes the lower explosive limit of the produced biomass gas; its unit is %. Index *k*_4_ shows the ability of ventilation; it is reflected by the air change rate and its unit is times/h. Index *k*_5_ reflects the ability of pressure relief when fire and explosion appear in the biomass gasification station; it was calculated by solving Eq 11 [48];

$$C=A/10V^{2/3}$$ (11)

where *C* denotes the pressure relief ratio (m^2^/m^3^), *A* denotes the area of pressure relief (m^2^), and *V* denotes the volume of the biomass gasification station (m^3^).

Index *k*_6_ shows the quantity of biomass materials that were stored in the biomass gasification station to produce biomass gas; its unit is m^3^.

Afterwards, the classification of hazard grade was given, as shown in Table 5; the classification rule was based on the related standard of PRC [48–50].

**Table 5:** Classification of hazard grade.

Index	Hazard Grade				
	I	II	III	IV	V
Biomass gas production rate (*k*_1_; m^3^/h)	100–500	500–1000	1000–3000	3000–5000	>5000
Volume fraction of CO (*k*_2_; %)	0–5	5–10	10–15	15–20	20–100
Lower explosive limit of biomass gas (*k*_3_; %)	100–30	30–20	20–15	15–10	10–0
Artificial ventilation atmosphere (*k*_4_; times/h)	>12	12–9	9–6	6–3	3–1
Pressure relief ratio (*k*_5_; m^2^/m^3^)	>0.25	0.25–0.2	0.2–0.16	0.16–0.11	0.11–0.03
Quantity of biomass materials (*k*_6_; m^3^)	0–10	10–5000	5000–10000	10000–50000	>50000

### Data Collection

The data related to the 6 indices were collected and calculated. The biomass gas production rate (*k*_1_) and the artificial ventilation atmosphere (*k*_4_) were the inherent properties of the biomass gasification station; the volume fraction of CO (*k*_2_) and the lower explosive limit of biomass gas (*k*_3_) can be confirmed by testing the produced biomass gas. However, the pressure relief ratio (*k*_5_) needed to be calculated based on the structure size of the biomass gasification station. Based on the corresponding standard of PRC [48], the area of pressure relief was equal to the area of windows and doors. According to the structure size, the area of pressure relief of Huangtukan station and Yanjia station was 17.22 m^2^ and 46.90 m^2^, respectively, while the volumes of the biomass gasification station of Huangtukan station and Yanjia station were 149.18 m^3^ and 278.43 m^3^, respectively. Then, the pressure relief ratio was calculated by solving Eq 11. Generally, the quantity of biomass materials (*k*_6_) was equal to one third of the volume of the biomass materials storage room; the volumes of the biomass materials storage room of Huangtukan station and Yanjia station were 40.62 m^3^ and 87.78 m^3^, respectively. Finally, the collected data were listed as shown in Table 6.

**Table 6:** Index data of Huangtukan station and Yanjia station.

Index	Huangtukan Station	Yanjia Station
Biomass gas production rate (*k*_1_; m^3^/h)	300	600
Volume fraction of CO (*k*_2_; %)	20.26	17.73
Lower explosive limit of biomass gas (*k*_3_; %)	18.47	21.98
Artificial ventilation atmosphere (*k*_4_; times/h)	10	8
Pressure relief ratio (*k*_5_; m^2^/m^3^)	0.0612	0.1110
Quantity of biomass materials (*k*_6_; m^3^)	13.54	29.26

## Results and Discussion

### Confirmation of Identity Degree, Discrepancy Degree and Contradistinction Degree

Based on the definition of SPA previously mentioned, the evaluations for each hazard grade, i.e., the values of identity degree, discrepancy degree and contradistinction degree, were confirmed by Tables 5 and 6; the results are listed in Table 7.

**Table 7:** Evaluations for each hazard grade.

Huangtukan Station					
	I(*a*)	II(*b*_1_)	III(*b*_2_)	IV(*b*_3_)	V(*c*)
*K*_1_	1	0	0	0	0
*K*_2_	0	0	0	0	1
*K*_3_	0	0	1	0	0
*K*_4_	0	1	0	0	0
*K*_5_	0	0	0	0	1
*K*_6_	0	1	0	0	0
Yanjia Station					
	I(*a*)	II(*b*_1_)	III(*b*_2_)	IV(*b*_3_)	V(*c*)
*K*_1_	0	1	0	0	0
*K*_2_	0	0	0	1	0
*K*_3_	0	1	0	0	0
*K*_4_	0	0	1	0	0
*K*_5_	0	0	0	1	0
*K*_6_	0	1	0	0	0

### Calculation of Cloud Connection Degree

**Fig 3:**
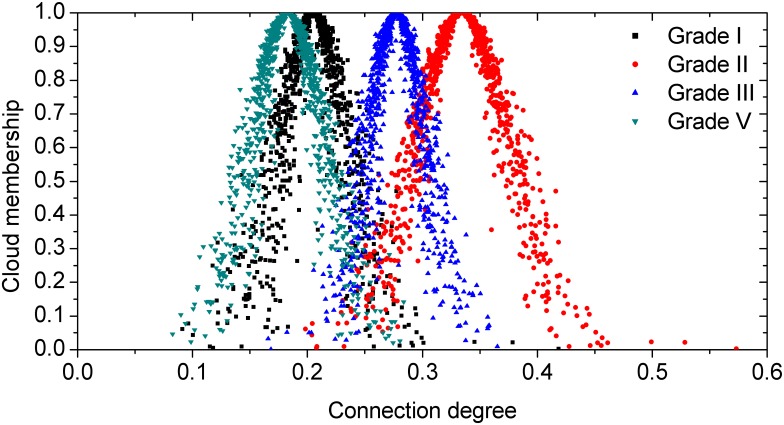
Normal clouds of CCD for Huangtukan station.

**Fig 4:**
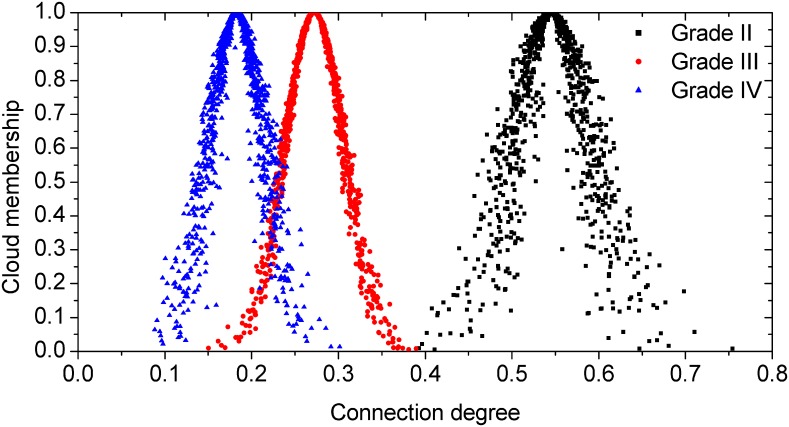
Normal clouds of CCD for Yanjia station.

After the evaluations for each hazard grade of the two stations were obtained, the CCD was calculated using Eq 9 through Eq 10 and Table 7 afterwards; the calculation results are listed in Table 8. Let the amounts of cloud drops be 1000; the corresponding normal clouds for each hazard grade were generated by the forward cloud generator and were as shown in Figs 3 and 4.

**Table 8:** Calculation results of CCD.

	Huangtukan Station	Yanjia Station
I	(0.2053, 0.0297, 0.0092)	0
II	(0.3344, 0.0415, 0.0080)	(0.5450, 0.0456, 0.0138)
III	(0.2776, 0.0258, 0.0072)	(0.2722, 0.0345, 0.0032)
IV	0	(0.1828, 0.0324, 0.0075)
V	(0.1828, 0.0324, 0.0075)	0

### Analysis of Hazard Assessment Results by CM-SPA

As shown in Figs 3 and 4, the calculated CCD of the hazard grade for Huangtukan station and Yanjia station clearly reflected the relationship of each hazard grade. Fig 3 shows that the expected value of the CD of grade II was the maximum. Hence, based on the maximal connection degree principle, as previously mentioned, the hazard grade of Huangtukan station mainly belonged to grade II. However, it can also be seen in Fig 3 that not all of the cloud drops of grade II were the maximum. Based on the '3 *En* rule', as previously mentioned, the CD of grades I, II, III and V were mainly focused on [0.1162, 0.2944], [0.2099, 0.4589], [0.2002, 0.3550] and [0.0856, 0.2800], respectively. It can be concluded that the intersection scope of grade II and grade III was the maximum; thus, the relevance of grade III to grade II was closer than for other grades. Then, it was summarized that the hazard grade of Huangtukan station was between grade II and grade III and closer to grade II. Hence, the hazard of Huangtukan station was between low hazard and middle hazard and closer to low hazard. On the other hand, as shown in Fig 4, the expected value and the whole cloud drops of the CD of grade II were both the maximum. Similarly, the CD of grades II, III and IV were mainly focused on [0.4082, 0.6818], [0.1687, 0.3757] and [0.0856, 0.2800] based on the '3 *En* rule', respectively. There was no intersection between grade II and the other grades. In other words, the hazard grade of Yanjia station completely belonged to grade II and the hazard of Yanjia station was low hazard.

For the hazard assessment results of Huangtukan station and Yanjia station, although the CD of grade II of the two stations both had the maximal expected value, regarding the difference of the distribution of cloud drops, it can be summarized that the hazard of the two stations was different.

### Comparison of Hazard Assessment Results by CM-SPA and AHP Based SPA

For the comparison and verification of hazard assessment results using CM-SPA, the hazard assessment using the traditional method of SPA was also made for biomass gasification stations. In regard to the traditional method of SPA, the assessment results are expressed as constant values due to indices weights were constant values. As AHP has been widely used in the confirmation of indices weights for SPA [32,33], thereby using AHP to confirm indices weights for the compared hazard assessment by SPA. In order to ensure the comparability, the data used in this paper were employed again to make hazard assessment by AHP based SPA. Owing to AHP was a classical approach [51] and the limited space in this paper, a brief introduction for the confirmation of indices weights obtained by AHP was given as followed [32].

1. Firstly, the hazard assessment for biomass gasification stations is set as the overall objective of AHP. Afterwards, accidents of fire, explosion and poisoning discussed in this paper are set to be the middle factors of AHP. Thereby setting the six assessment indices as the criteria of AHP.
2. The pair-wise comparisons [32] are used to make judgments for the importance of criteria to middle factors and middle factors to the overall objective, then the obtained pair-wise comparison is reflected by the judgments matrix. Herein, the 1/9-9 scale [33] is used to make comparison. Therefore, the judgments matrix for fire, explosion and poisoning to the overall objective are shown as the matrix *M*_1_ (Eq 12), and the judgments matrices for the six assessment indices to fire, explosion and poisoning are shown by matrices *M*_2_, *M*_3_ and *M*_4_ (Eqs 13–15), respectively.
  $$M_{1}=\left[\begin{array}{ccc} \alpha_{p_{1},p_{1}} & \alpha_{p_{1},p_{2}} & \alpha_{p_{1},p_{3}}\\ \alpha_{p_{2},p_{1}} & \alpha_{p_{2},p_{2}} & \alpha_{p_{2},p_{3}}\\ \alpha_{p_{3},p_{1}} & \alpha_{p_{3},p_{2}} & \alpha_{p_{3},p_{3}} \end{array}\right]=\left[\begin{array}{ccc} 1 & 1/3 & 1\\ 3 & 1 & 2\\ 1 & 1/2 & 1 \end{array}\right]$$ (12)
  where *p*_1_, *p*_2_ and *p*_3_ denote the fire, explosion and poisoning, respectively, *α* denotes the pair-wise comparison result.
  $$M_{2}=\left[\begin{array}{cccc} \alpha_{k_{1},k_{1}} & \alpha_{k_{1},k_{3}} & \alpha_{k_{1},k_{4}} & \alpha_{k_{1},k_{6}}\\ \alpha_{k_{3},k_{1}} & \alpha_{k_{3},k_{3}} & \alpha_{k_{3},k_{4}} & \alpha_{k_{3},k_{6}}\\ \alpha_{k_{4},k_{1}} & \alpha_{k_{4},k_{3}} & \alpha_{k_{4},k_{4}} & \alpha_{k_{4},k_{6}}\\ \alpha_{k_{6},k_{1}} & \alpha_{k_{6},k_{3}} & \alpha_{k_{6},k_{4}} & \alpha_{k_{6},k_{6}} \end{array}\right]=\left[\begin{array}{cccc} 1 & 1/3 & 1/2 & 1\\ 3 & 1 & 2 & 4\\ 2 & 1/2 & 1 & 2\\ 1 & 1/4 & 1/2 & 1 \end{array}\right]$$ (13)
  $$M_{3}=\left[\begin{array}{cccc} \alpha_{k_{1},k_{1}} & \alpha_{k_{1},k_{3}} & \alpha_{k_{1},k_{4}} & \alpha_{k_{1},k_{5}}\\ \alpha_{k_{3},k_{1}} & \alpha_{k_{3},k_{3}} & \alpha_{k_{3},k_{4}} & \alpha_{k_{3},k_{5}}\\ \alpha_{k_{4},k_{1}} & \alpha_{k_{4},k_{3}} & \alpha_{k_{4},k_{4}} & \alpha_{k_{4},k_{5}}\\ \alpha_{k_{5},k_{1}} & \alpha_{k_{5},k_{3}} & \alpha_{k_{5},k_{4}} & \alpha_{k_{5},k_{5}} \end{array}\right]=\left[\begin{array}{cccc} 1 & 1/4 & 1/2 & 1/3\\ 4 & 1 & 2 & 3\\ 2 & 1/2 & 1 & 2\\ 3 & 1/3 & 1/2 & 1 \end{array}\right]$$ (14)
  $$M_{4}=\left[\begin{array}{ccc} \alpha_{k_{1},k_{1}} & \alpha_{k_{1},k_{2}} & \alpha_{k_{1},k_{4}}\\ \alpha_{k_{2},k_{1}} & \alpha_{k_{2},k_{2}} & \alpha_{k_{2},k_{4}}\\ \alpha_{k_{4},k_{1}} & \alpha_{k_{4},k_{2}} & \alpha_{k_{4},k_{4}} \end{array}\right]=\left[\begin{array}{ccc} 1 & 3 & 1/2\\ 1/3 & 1 & 1/4\\ 2 & 4 & 1 \end{array}\right]$$ (15)
3. Finally, indices weights are calculated based on the above matrices while calculation results are listed in Table 9.

**Table 9:** Indices weights obtained by AHP.

	*k*_1_	*k*_2_	*k*_3_	*k*_4_	*k*_5_	*k*_6_
Weight	0.1586	0.0293	0.3594	0.3273	0.0985	0.0269

After the AHP based indices weights were obtained, the CDs were then computed based on Eq 3, Tables 7 and 9. Calculation results, i.e., hazard assessment results by SPA are shown in Table 10.

**Table 10:** Calculation results of CD.

	Huangtukan Station	Yanjia Station
I	0.1586	0
II	0.3542	0.5449
III	0.3594	0.3273
IV	0	0.1278
V	0.1278	0

According to the maximal connection degree principle as previously mentioned, it can concluded that the hazard grades of Huangtukan station and Yanjia station were grade III (middle hazard) and grade II (low hazard), respectively. Obviously, the obtained hazard assessment results by AHP based SPA weren't reasonable and precise enough due to the influences of other parameters of the CD were neglected. For example, in regard to the hazard assessment result of Huangtukan station, though assessment values of grade II (0.3542) and grade III (0.3594) were almost equal, the hazard assessment result can only confirmed to be grade III due to the maximal connection degree principle. In addition, with the exception of the maximal parameter of the CD, some other parameters weren't considered in confirming hazard assessment results even though values of them weren't '0'.

By contrast with CM-SPA, the hazard assessment results obtained by the traditional method of SPA can only be reflected by the stationary hazard grade due to calculated indices weights were constant values. Thus the subjectivity of the confirmation for indices weights will vastly affect hazard assessment results. For the CM-SPA, further and complete hazard assessments will be made based on the randomness and fuzziness of CM; the assessment results will be more precise and reasonable. To sum up, CM-SPA could be a more effective and scientific method for hazard assessment of a biomass gasification station, in contrast with SPA.

## Conclusions

Because various hazardous factors exist in a biomass gasification system, hazard assessment is needed to evaluate the hazard degree of a biomass gasification station. In this study, a novel hazard assessment method was proposed based on CM-SPA. After a study of the method, conclusions were summarized and are listed below.

1. CM was employed to improve SPA. The weight of index was replaced by the proposed cloud weight in this study. In contrast with the traditional weight of index, the cloud weight was defined as the cloud descriptors and can reflect the randomness and fuzziness of experts judgments.
2. CCD was proposed and used to confirm the hazard grade of a biomass gasification station instead of CD; meanwhile, the calculation algorithm of CCD was worked out. Hence, the hazard assessment results were shown as cloud descriptors and each hazard grade was related to a corresponding normal cloud. Then, hazard assessment of a biomass gasification station was made via the analysis of the cloud descriptors in CM. Based on the randomness and fuzziness of CM, the assessment results will be more reasonable and scientific.
3. Two biomass gasification stations in Shenyang City, Liaoning Province, Northeast China, were made hazard assessment by CM-SPA and AHP based SPA, respectively. The comparison of hazard assessment results illustrated that the CM-SPA was a more effective, reasonable and scientific method for the hazard assessment of a biomass gasification station.
